# Effect of proteinuria at relapse on shear wave velocity assessed using ultrasound elastography in children with idiopathic nephrotic syndrome

**DOI:** 10.1007/s10396-024-01455-7

**Published:** 2024-04-13

**Authors:** Tomohiko Nishino, Shinya Tomori, Sayaka Ono, Kazuhiro Takahashi, Masakazu Mimaki

**Affiliations:** Department of Pediatrics, Teikyo University School of Medicine, 2-11-1 Kaga, Itabashi-Ku, Tokyo, 173-8605 Japan

**Keywords:** Elasticity imaging techniques, Nephrotic syndrome, Proteinuria, Relapse, Ultrasonography

## Abstract

**Purpose:**

Shear wave velocity (SWV) is an ultrasound elastography technique that provides much information for kidney disease assessment. However, the factors that alter SWV are not fully understood; it is unclear whether the variation in SWV seen in proteinuria associated with disease progression is due to tissue or proteinuria. This study investigated the effect of proteinuria on SWV.

**Methods:**

This prospective observational study compared SWV at remission with SWV at relapse in children treated for idiopathic nephrotic syndrome (INS) between April 2020 and December 2023. All relapses without oral steroids during the observation period were measured. SWV at remission was defined as the date closest to relapse during which repeated measurements were taken approximately every 3 months after steroid discontinuation.

**Results:**

Eight patients were treated for INS with a median observation period of 21.9 months (11.8–27.1). Of the 15 relapses, five that met the definition were considered for the study. The median interval between the measurement at relapse and remission was 40 days (11–55). SWV was significantly lower at relapse than remission (2.40 ± 0.20 m/s vs. 2.14 ± 0.15 m/s, *P* < 0.01).

**Conclusions:**

SWV decreased in the presence of severe proteinuria at relapse compared to the remission measurements. Although more cases need to be studied, the decrease in SWV may reflect the mechanism by which protein leaks into the urine, not just a direct change caused by the presence of proteinuria.

## Introduction

Ultrasound elastography consists of noninvasively measuring tissue hardness as a biomarker of disease. It can be used to evaluate disease progression and pathology. Among them, shear wave elastography uses the property of propagation velocity, which increases with the degree of tissue hardness, and is effective in evaluating the condition of various organs [1]. This method has been applied to nephrology, and by focusing on fibrosis, some insight has been gained regarding various conditions, such as chronic kidney disease (CKD), kidney transplantation, and glomerular diseases, including nephrotic syndrome (NS) [2–4]. However, some of these studies have produced contradictory reports, and there remain numerous unanswered questions regarding the use of this method in nephrology [5, 6]. It is unclear what the obtained measurements depend on as the kidney has different characteristics compared to other organs in terms of structure, morphology, and blood flow [7, 8]. Thus, to accurately interpret ultrasound elastography of the kidney, each of the factors influencing the measurements needs to be considered.

Proteinuria is a highly prevalent symptom in several kidney diseases. Studies on proteinuria and ultrasound elastography in the literature are related to CKD and kidney transplantation [5]. However, since proteinuria appears with progressive kidney fibrosis in these diseases, ultrasound elastography measurements may not depend solely on proteinuria. Accordingly, it is necessary to elucidate the effect of proteinuria alone on ultrasound elastography measurements.

The mechanism of proteinuria in pediatric idiopathic NS (INS) differs from the mechanism of kidney disease associated with progressive kidney fibrosis [9]. Approximately 80% of pediatric INS cases have minimal change NS (MCNS) without glomerulosclerosis, and more than 90% of them are steroid-sensitive [10]. The selectivity index (SI: the clearance ratio of IgG to transferrin) is an established parameter used for differentiating such kidney tissues. The charge and size of the leaking protein reflect the kidney tissue; thus, if the protein is highly selective (i.e., its SI is low), MCNS is probable [11]. Steroid-sensitive NS (SSNS) is generally treated as synonymous with MCNS in pediatrics [12]. No kidney tissue changes are observed during the remission period without proteinuria in this disease. Typical INS management consists of daily checks during the remission period using the urine dipstick test to ensure that relapse can be detected before osmotic edema. Therefore, the influence of factors such as tissue changes, hemodynamics, and edema can be minimized as much as possible during ultrasound elastography measurement at the time of relapse in pediatric INS, making it suitable for evaluating proteinuria only.

Imaging evaluation is important for early detection, monitoring, and assessment of disease progression and prognosis [13]. Understanding the factors that influence the measurements will improve the prognosis of patients with kidney disease. The purpose of this study was to investigate the effect of proteinuria on shear wave velocity (SWV), a form of ultrasound elastography.

## Materials and methods

This prospective observational study was designed to compare SWV at remission without proteinuria and SWV at relapse with severe proteinuria in pediatric INS. Participation in this study did not require any change in treatment or management protocol for the patients.

### Study population

Children aged < 16 years who received treatment for INS at Teikyo University Hospital in Japan between April 1, 2020 and December 31, 2023 were included in the study. The diagnosis of INS and definition of relapse and remission were decided according to the clinical guidelines issued by the Japanese Society for Pediatric Nephrology [10]; secondary NS was excluded.

In pediatric INS treatment, alternate-day administration is widely used as the standard protocol for steroid reduction [14]. Because steroids affect the kidney blood flow and mineral balance [15], the potential for steroids to alter SWV cannot be ruled out. INS relapse occurs unexpectedly, making it difficult to control the date and dose of steroid administration at relapse. Therefore, relapses during steroid administration were excluded.

### Treatment

The treatment was administered according to the guidelines in Japan [10]. Specifically, the steroid dose was tapered over a period of 3–6 months during remission after relapse, and immunosuppressive drugs were added in cases of frequently relapsing NS (FRNS) and/or steroid-dependent NS (SDNS).

### Timing of SWV measurement at remission and relapse (Fig. 1)

**Fig. 1:**
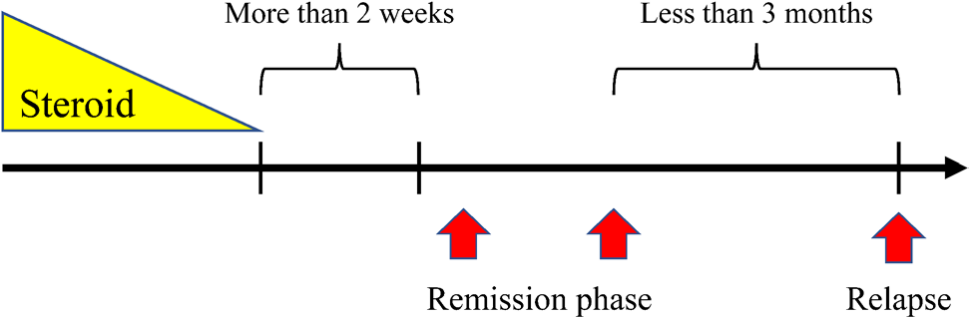
Timing of measurements. The arrow from left to right represents the passage of time. The top row shows the steroid dosage tapering and the pertinent time periods. The bottom row shows the stages of the disease at which measurements were performed in this study. SWV was measured repeatedly during the remission period, and the measurement closest to the time of relapse was considered for the study

All measurements were performed midmorning with at least 1 h of nil per os.

SWV at remission: the measurement was started 2 weeks after the cessation of steroid medication and repeated approximately every 3 months, and the measurement closest to the date of relapse was selected.

SWV at relapse: the measurement performed on the first or second day of the three consecutive days of ≥ 3 + protein on the urine dipstick test at the first instance of urination in the morning.

### Measurement method for SWV

**Fig. 2:**
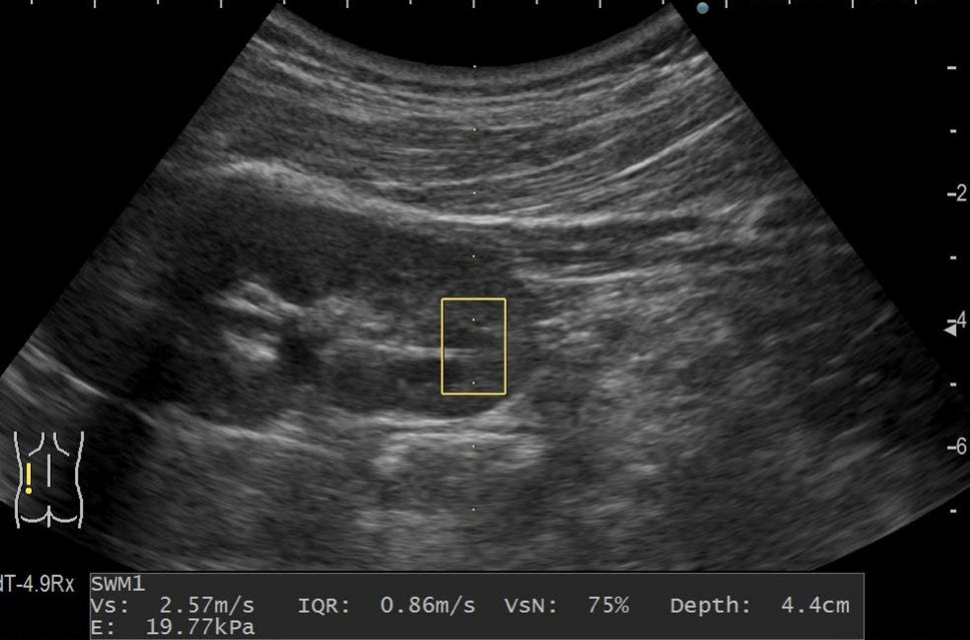
Region of interest (ROI) setting and measurement method of shear wave velocity (SWV)**.** The selected ROI (10 × 15 mm) surrounded by a yellow line was set at the site of the cortex of the lower pole of the left kidney, vertically below the probe. Measurements were performed by lightly applying the probe on the back of the reclining patient, with as little manual pressure drainage as possible. The result of the SWV measurement was output as Vs, and other values including VsN were shown at the same time as the measurement result. *Vs* shear wave velocity, *IQR* interquartile range, *VsN* Vs effective rate, *E* elastic modulus

Measurements were performed by a single pediatric nephrologist. For measurement, the older children were placed in the supine position on the bed, and the younger children were held by their parents. The patients were instructed to relax and breathe naturally. All measurements were acquired posteriorly, and the region of interest (ROI) was set at the lower pole, excluding the great vessels and kidney pelvis whenever possible (Fig. 2). Since our facility usually performs kidney biopsies on the right kidney, SWV measurements were standardized to the left kidney. The modality used was an ultrasound system (ARIETTA 70; Hitachi Aloka Medical, Japan) with a convex probe (1–5 MHz).

### Statistical analyses

All statistical analyses were performed using R version 3.6.1 (The R Foundation for Statistical Computing, Vienna, Austria). The net amount of effective SWV (SWV effective rate: VsN) was used in this study to ensure the reliability of the SWV values, because the VsN values were displayed at the same time as the measurement, removing measurer bias. Since reliability can be maintained when VsN is ≥ 50 [16], this criterion was also selected in this study. The mean values were calculated for each SWV measurement, and these were compared between remission and relapse using paired *t *test. Statistical significance was set at *P* < 0.05.

## Results

### Study population

The initial characteristics of the eight patients diagnosed with INS at the time of their presentation are shown in Table 1. They had no congenital anomalies of the kidney and urinary tract or family with kidney disease. The median time observed was 21.9 months (11.8–27.1). The clinical course during the study period included all patients with a steroid-sensitive comorbidity: four patients with SDNS, without transition to steroid-resistant NS. All patients with SDNS underwent kidney biopsy related to cyclosporine A (CsA) treatment and were diagnosed with MCNS based on the result.

**Table 1:** Patient characteristics at initial onset

	Sex	Age at initial onset (years)	Selectivity index	Time from initial onset to remission (days)
Patient 1	Boy	2.0	0.07	11
Patient 2	Boy	2.3	0.05	6
Patient 3	Girl	2.8	0.01	9
Patient 4	Boy	6.8	0.65	7
Patient 5	Boy	6.9	0.08	8
Patient 6	Boy	8.3	0.15	10
Patient 7	Boy	12.0	0.12	6
Patient 8	Boy	14.6	0.23	12

### Relapse

During the observation period, 10 out of 15 relapses were excluded, because the patient was on steroids, and five relapses were considered for the study. No relapse of cases that had been vaccinated immediately before relapse or cases of common cold or fever at the time of relapse were observed. The median interval between measurement at relapse and that at remission was 40 (11–55) days. In one of the five relapses, CsA was administered, and there was no difference in the dose between remission and relapse. No medications other than CsA were administered at any instance of measurement.

### SWV measurements

**Fig. 3:**
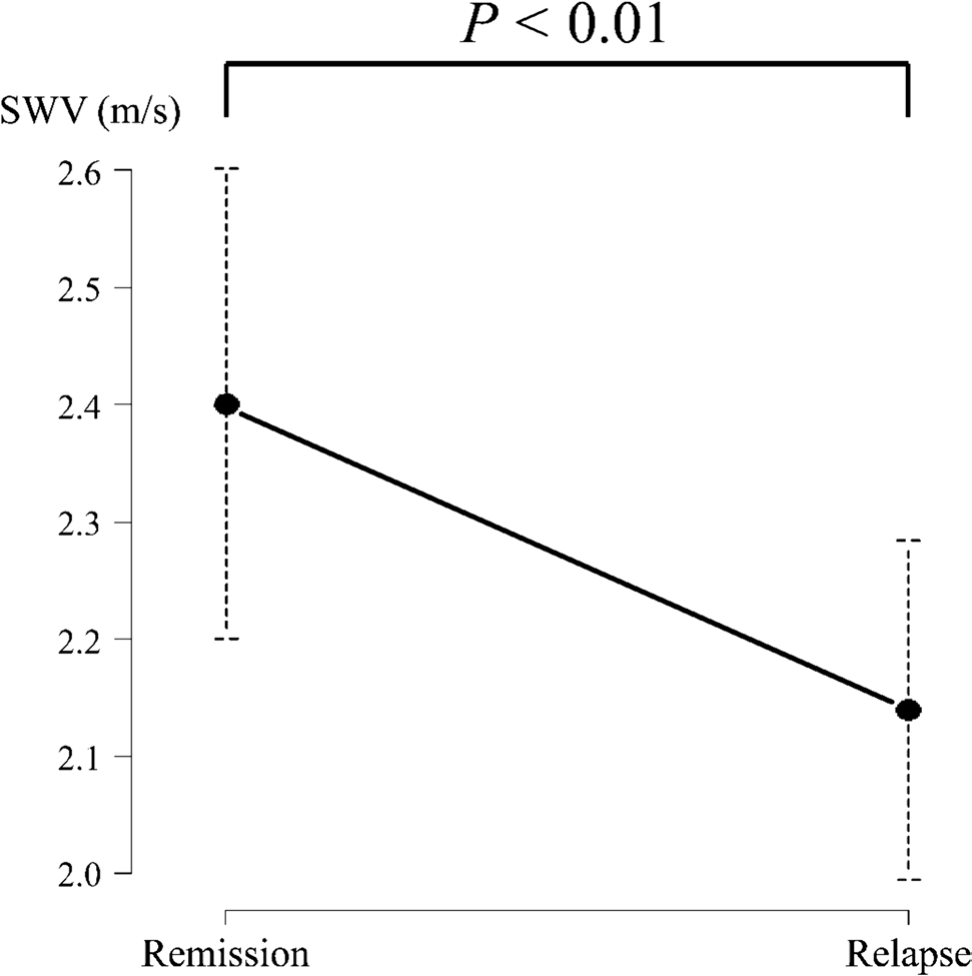
Shear wave velocity (SWV) values at remission and relapse. The vertical axis represents the SWV (m/s), and the horizontal axis represents the stage of the disease at which the measurement was acquired. SWV was significantly lower at relapse than that at remission (*P* < 0.01)

SWV was measured ≥ 45 times in each examination in the same position at remission and at relapse. The SWV results for each measurement are shown in Table 2. SWV was significantly lower at relapse than at remission (2.40 ± 0.20 m/s vs. 2.14 ± 0.15 m/s, *P* < 0.01) (Fig. 3).

**Table 2:** Shear wave velocity, age, and urinalysis findings at the time of each measurement

	No	Age at relapse (years)	Interval between remission and relapse (days)	U-PCR at remission (g/gCr)	U-PCR at relapse (g/gCr)	Mean SWV (m/s)	
						Remission	Relapse
Patient 2	1	2.6	11	0.15	2.94	2.31 ± 0.26	2.04 ± 0.37
	2	3.5	55	0.05	1.22	2.37 ± 0.36	2.02 ± 0.26
Patient 7	3	12.5	40	0.03	1.70	2.19 ± 0.40	2.04 ± 0.46
	4	12.9	10	0.17	1.05	2.40 ± 0.50	2.27 ± 0.43
Patient 8	5	15.7	78	0.05	2.63	2.73 ± 0.52	2.32 ± 0.31

*U-PCR* urine protein/creatinine ratio, *HPF* high-power field, *SWV* shear wave velocity

## Discussion

In this study, factors that affect SWV other than proteinuria were excluded as far as possible. SWV decreased significantly as proteinuria occurred. The decrease in SWV shown in this study provides a completely new insight. To the best of our knowledge, no study has focused solely on the effect of proteinuria on SWV. Understanding this effect will be of great benefit in the evaluation of NS and other kidney diseases.

The decrease in SWV may reflect the changes in the glomerulus, where proteinuria occurs in INS. In recent years, studies on many hereditary proteinuria syndromes have made important contributions to our knowledge regarding normal glomerular filter function and the mechanisms of proteinuria [17, 18]. Proteinuria in MCNS is considered to be the result of defects in the glomerular filtration barrier [19], such as glomerular permeability factors [20], podocytes [21], nephrin [22, 23], and loss of charge due to activation of CD80 in the basement membrane [24]. In the slit diaphragm structure of the glomerulus, molecular changes are induced, and protein leakage occurs [9, 25]. As a result of these changes, the glomerular filtration rate decreases during recurrent episodes of severe proteinuria in MCNS [26]. Therefore, the decrease in SWV observed in this study may have resulted from the decrease in the glomerular filtration rate that occurs in the proteinuria process. This new knowledge may help explain the role of the molecular mechanism of proteinuria, and we await confirmation of this through investigation of more cases going forward.

The process through which proteinuria occurs may have a stronger effect on SWV than that for proteinuria itself. Remarkably, SWV decreased at the time of relapse with massive proteinuria in this study. In contrast, the previous reports have shown a significant positive correlation between albuminuria or proteinuria and ultrasound elastography results [27–30]. The participants of these previous studies had diabetic nephropathy (DN) and kidney transplantation with kidney fibrosis, and the mechanism underlying the occurrence of proteinuria differs in pediatric INS. In DN, proteinuria occurs due to increased glomerular capillary pressure, cellular hypertrophy, accumulation of extracellular matrix with fibrous collagen, and endothelial cell damage caused by the activity of the renin-angiotensin system [31]. This same change was histologically observed in a rat model of CKD [32]. Additionally, glomerular loss due to these diseases causes hyperfiltration in the remaining glomeruli [33]. This hyperfiltration may increase osmolarity [34], resulting in higher SWV due to increased intraglomerular pressure. These differences in the processes involved in the pathogenesis of proteinuria may be the cause of the variation in SWV, contrary to previously reported results.

The effect on SWV is more strongly reflected by circulatory dynamics and tissue fibrosis than by proteinuria. There have been several reports on the use of ultrasound elastography for INS. A previous study of 120 adult patients with INS showed that SWV was significantly higher at the initial onset before treatment than that in 30 healthy controls [35]. However, this previous study differed from the present study in that the blood albumin levels were clearly lower in the INS group, because it was measured at the time of initial disease onset. It is known that SWV largely reflects blood flow and blood pressure in kidney ultrasound elastography [36–38]; thus, the effect of perfusion masks other factors [39]. Therefore, it is possible that high SWV during severe proteinuria at the initial onset of INS in this previous study could be attributed to the effect of oncotic pressure. In addition, a report comparing 90 adult patients with INS and 30 healthy controls showed that SWV was positively correlated with the percentage of interstitial lesions in patients with moderate and severe cases of the disease [40]. The previous report included chronic nephritis in its scope, because the disease concept of INS is different from that in children; it is difficult to compare it directly with this study. All children in this study showed relatively low SI readings and experienced SSNS. The different results shown in the previous reports, such as the increase in SWV, indirectly support the strong influence of factors other than proteinuria. At the time of relapse in this study, at least 3 months had passed since the steroid tapering period from the disappearance of proteinuria; therefore, it is unlikely that hypoproteinemia was present, although this was not confirmed through blood tests. Additionally, it is unlikely that kidney tissue will undergo changes such as fibrosis during the remission period without proteinuria. In general, aging during childhood and disease progression increase SWV [41, 42]. Therefore, proteinuria in INS decreases SWV, but this variability may be masked by other factors to a certain degree.

There were several limitations to our findings. First, the ROI settings varied because of age-dependent body size and respiration. In this study, the kidney pelvis and great vessels were excluded from the ROI as much as possible; however, portions of it were included. It has long been known that establishing ROI settings in children is challenging [42, 43], and measurement at the same site is recommended [44]. In this study, only the left kidney lower pole was measured in all instances. Although it may not be valid to compare the absolute values obtained in this study with those of other reports, they are considered reliable in terms of the observed SWV change as the same site was consistently measured. Second, the sample size was small due to the single-center experience. However, using a single measurer eliminated measurement bias, and having the pediatric nephrologist who usually communicated with the children perform the measurements stabilized the test. Although the number of patients was small, it is worth noting that SWV decreased in all relapses. It is hoped that future studies with a larger number of patients will improve our understanding of proteinuria.

## Conclusion

SWV significantly decreased when severe proteinuria occurred during relapse in this study, in which factors other than proteinuria that affect SWV were removed as much as possible in pediatric INS. It was inferred that this variation in SWV is strongly related to the mechanism of protein leakage into the urine. Although more cases should be investigated in the future, understanding how proteinuria occurs in many kidney diseases clarifies the role of ultrasound elastography in kidney diseases.

## Data Availability

The datasets generated during and/or analyzed in the present study are available from the corresponding author on reasonable request.

## References

[1] Ozturk A, Grajo JR, Dhyani M, et al. Principles of ultrasound elastography. Abdom Radiol (NY). 2018;43:773–85. 29487968 10.1007/s00261-018-1475-6 PMC5973820

[2] Sigrist RMS, Liau J, Kaffas AE, et al. Ultrasound elastography: review of techniques and clinical applications. Theranostics. 2017;7:1303–29. 28435467 10.7150/thno.18650 PMC5399595

[3] Yogurtcuoglu B, Damar C. Renal elastography measurements in children with acute glomerulonephritis. Ultrasonography. 2021;40:575–83. 33906284 10.14366/usg.20173 PMC8446499

[4] Shi LQ, Sun J, Yuan L, et al. Diagnostic performance of renal cortical elasticity by supersonic shear wave imaging in pediatric glomerular disease. Eur J Radiol. 2023;168:111113. 37820521 10.1016/j.ejrad.2023.111113

[5] Lim WTH, Ooi EH, Foo JJ, et al. shear wave elastography: a review on the confounding factors and their potential mitigation in detecting chronic kidney disease. Ultrasound Med Biol. 2021;47:2033–47. 33958257 10.1016/j.ultrasmedbio.2021.03.030

[6] Ce M, Felisaz PF, Ali M, et al. Ultrasound elastography in chronic kidney disease: a systematic review and meta-analysis. J Med Ultrason. 2023;50:381–415. 10.1007/s10396-023-01304-z 37186192

[7] Grenier N, Poulain S, Lepreux S, et al. Quantitative elastography of renal transplants using supersonic shear imaging: a pilot study. Eur Radiol. 2012;22:2138–46. 22588518 10.1007/s00330-012-2471-9

[8] Jiang K, Ferguson CM, Lerman LO. Noninvasive assessment of renal fibrosis by magnetic resonance imaging and ultrasound techniques. Transl Res. 2019;209:105–20. 31082371 10.1016/j.trsl.2019.02.009 PMC6553637

[9] Cara-Fuentes G, Clapp WL, Johnson RJ, et al. Pathogenesis of proteinuria in idiopathic minimal change disease: molecular mechanisms. Pediatr Nephrol. 2016;31:2179–89. 27384691 10.1007/s00467-016-3379-4

[10] Ishikura K, Matsumoto S, Sako M, et al. Clinical practice guideline for pediatric idiopathic nephrotic syndrome 2013: medical therapy. Clin Exp Nephrol. 2015;19:6–33. 25653046 10.1007/s10157-014-1030-x

[11] Laurent J, Philippon C, Lagrue G, et al. Proteinuria selectivity index–prognostic value in lipoid nephrosis and related diseases. Nephron. 1993;65:185–9. 8247178 10.1159/000187472

[12] Vivarelli M, Massella L, Ruggiero B, et al. Minimal change disease. Clin J Am Soc Nephrol. 2017;12:332–45. 27940460 10.2215/CJN.05000516 PMC5293332

[13] Viteri B, Calle-Toro JS, Furth S, et al. State-of-the-art renal imaging in children. Pediatrics. 2020;145:e20190829. 31915193 10.1542/peds.2019-0829 PMC6993529

[14] Gipson DS, Massengill SF, Yao L, et al. Management of childhood onset nephrotic syndrome. Pediatrics. 2009;124:747–57. 19651590 10.1542/peds.2008-1559

[15] Kleeman CR, Levi J, Better O. Kidney and adrenocortical hormones. Nephron. 1975;15:261–78. 1101088 10.1159/000180516

[16] Yada N, Sakurai T, Minami T, et al. A newly developed shear wave elastography modality: with a unique reliability index. Oncology. 2015;89(Suppl 2):53–9. 26580548 10.1159/000440632

[17] Haraldsson B, Nystrom J, Deen WM. Properties of the glomerular barrier and mechanisms of proteinuria. Physiol Rev. 2008;88:451–87. 18391170 10.1152/physrev.00055.2006

[18] Tryggvason K, Patrakka J, Wartiovaara J. Hereditary proteinuria syndromes and mechanisms of proteinuria. N Engl J Med. 2006;354:1387–401. 16571882 10.1056/NEJMra052131

[19] Carrie BJ, Salyer WR, Myers BD. Minimal change nephropathy: an electrochemical disorder of the glomerular membrane. Am J Med. 1981;70:262–8. 6162382 10.1016/0002-9343(81)90760-9

[20] Cho MH, Hong EH, Lee TH, et al. Pathophysiology of minimal change nephrotic syndrome and focal segmental glomerulosclerosis. Nephrology (Carlton). 2007;12(Suppl 3):S11–4. 17995521 10.1111/j.1440-1797.2007.00875.x

[21] Tojo A. Mechanism underlying selective albuminuria in minimal change nephrotic syndrome. Int J Nephrol. 2019;2019:5859102. 31781392 10.1155/2019/5859102 PMC6874928

[22] Doublier S, Ruotsalainen V, Salvidio G, et al. Nephrin redistribution on podocytes is a potential mechanism for proteinuria in patients with primary acquired nephrotic syndrome. Am J Pathol. 2001;158:1723–31. 11337370 10.1016/S0002-9440(10)64128-4 PMC1891937

[23] Wernerson A, Duner F, Pettersson E, et al. Altered ultrastructural distribution of nephrin in minimal change nephrotic syndrome. Nephrol Dial Transplant. 2003;18:70–6. 12480962 10.1093/ndt/18.1.70

[24] Kaneko K, Tsuji S, Kimata T, et al. Pathogenesis of childhood idiopathic nephrotic syndrome: a paradigm shift from T-cells to podocytes. World J Pediatr. 2015;11:21–8. 25822700 10.1007/s12519-015-0003-9

[25] Lahdenkari AT, Lounatmaa K, Patrakka J, et al. Podocytes are firmly attached to glomerular basement membrane in kidneys with heavy proteinuria. J Am Soc Nephrol. 2004;15:2611–8. 15466265 10.1097/01.ASN.0000139478.03463.D9

[26] Myers BD, Guasch A. Mechanisms of proteinuria in nephrotic humans. Pediatr Nephrol. 1994;8:107–12. 8142207 10.1007/BF00868285

[27] Sumbul HE, Koc AS, Gulumsek E. Renal cortical stiffness is markedly increased in pre-diabetes mellitus and associated with albuminuria. Singap Med J. 2020;61:435–42. 10.11622/smedj.2019052 PMC7926591 31197376

[28] Gungor O, Guzel FB, Sarica MA, et al. Ultrasound elastography evaluations in patient populations with various kidney diseases. Ultrasound Q. 2019;35:169–72. 30601446 10.1097/RUQ.0000000000000404

[29] Fang JX, Chen XY, Yang QM, et al. Factors Influencing renal parenchymal stiffiness in patients with diabetic nephropathy. Int J Gen Med. 2021;14:1911–7. 34040423 10.2147/IJGM.S311420 PMC8140885

[30] Ruan Z, Xiao Z, Shi X, et al. Comparison of sound touch elastography and quantification for assessing the renal pathologic changes in patients with proteinuria. Insights Imaging. 2023;14:135. 37541990 10.1186/s13244-023-01476-9 PMC10403462

[31] Burns KD. Angiotensin II and its receptors in the diabetic kidney. Am J Kidney Dis. 2000;36:449–67. 10977776 10.1053/ajkd.2000.16192

[32] Derieppe M, Delmas Y, Gennisson JL, et al. Detection of intrarenal microstructural changes with supersonic shear wave elastography in rats. Eur Radiol. 2012;22:243–50. 21845464 10.1007/s00330-011-2229-9

[33] Brenner BM. Nephron adaptation to renal injury or ablation. Am J Physiol. 1985;249:F324–37. 3898871 10.1152/ajprenal.1985.249.3.F324

[34] Schnaper HW. Remnant nephron physiology and the progression of chronic kidney disease. Pediatr Nephrol. 2014;29:193–202. 23715783 10.1007/s00467-013-2494-8 PMC3796124

[35] Yang X, Hou FL, Zhao C, et al. The role of real-time shear wave elastography in the diagnosis of idiopathic nephrotic syndrome and evaluation of the curative effect. Abdom Radiol (NY). 2020;45:2508–17. 32107581 10.1007/s00261-020-02460-3

[36] Asano K, Ogata A, Tanaka K, et al. Acoustic radiation force impulse elastography of the kidneys: is shear wave velocity affected by tissue fibrosis or renal blood flow? J Ultrasound Med. 2014;33:793–801. 24764334 10.7863/ultra.33.5.793

[37] Mocnik M, Golob Jancic S, Marcun VN. Liver and kidney ultrasound elastography in children and young adults with hypertension or chronic kidney disease. Pediatr Nephrol. 2023;38:3379–87. 37154960 10.1007/s00467-023-05984-0

[38] Bruce-Hickman D, Lim ZY, Lim HY, et al. Measurement of renal congestion and compliance following intravenous fluid administration using shear wave elastography. Crit Care Resusc. 2023;25:27–32. 37876990 10.1016/j.ccrj.2023.04.006 PMC10581263

[39] Grossmann M, Tzschatzsch H, Lang ST, et al. US time-harmonic elastography for the early detection of glomerulonephritis. Radiology. 2019;292:676–84. 31287390 10.1148/radiol.2019182574

[40] Yang X, Yu N, Yu J, et al. Virtual touch tissue quantification for assessing renal pathology in idiopathic nephrotic syndrome. Ultrasound Med Biol. 2018;44:1318–26. 29650267 10.1016/j.ultrasmedbio.2018.02.012

[41] Leong SS, Wong JHD, Md Shah MN, et al. Shear wave elastography accurately detects chronic changes in renal histopathology. Nephrology (Carlton). 2021;26:38–45. 33058334 10.1111/nep.13805

[42] Lee MJ, Kim MJ, Han KH, et al. Age-related changes in liver, kidney, and spleen stiffness in healthy children measured with acoustic radiation force impulse imaging. Eur J Radiol. 2013;82:e290–4. 23433651 10.1016/j.ejrad.2013.01.018

[43] Karaman ZF, Kardas F. Determining the effects of excess weight on renal cortical stiffness in children and adolescents with point shear wave elastography. Med Ultrason. 2021;23:271–6. 33793699 10.11152/mu-2855

[44] Peride I, Radulescu D, Niculae A, et al. Value of ultrasound elastography in the diagnosis of native kidney fibrosis. Med Ultrason. 2016;18:362–9. 27622414 10.11152/mu.2013.2066.183.per

